# Preliminary Clinical Outcomes of Culturally Adapted Cognitive Behavioral Therapy for Reducing Panic Disorder Symptoms and Improving Quality of Life: Pilot Study

**DOI:** 10.3390/bs16091682

**Published:** 2026-09-18

**Authors:** Jamil Nasif, Agnes Chong Shu Sze, Mahadir Ahmad

**Affiliations:** Center for Community Health Studies (ReaCH), Faculty of Health Sciences, Universiti Kebangsaan Malaysia, Kuala Lumpur 50300, Malaysia; mahadir@ukm.edu.my

**Keywords:** panic disorder, cognitive behavioral therapy, cultural adaptation, pilot randomized study, quality of life

## Abstract

Cognitive behavioral therapy (CBT) is an evidence-based treatment for panic disorder, but culturally adapted interventions remain insufficiently studied in Palestinian populations. Pilot trials are needed to estimate recruitment, retention, acceptability, and preliminary clinical outcomes before a definitive trial is undertaken. To examine preliminary clinical outcomes of a culturally adapted cognitive behavioral therapy (CA-CBT) intervention for reducing panic disorder symptoms and improving quality of life among Palestinian adults. A two-arm randomized pilot study with a waitlist control was conducted among 30 adults diagnosed with panic disorder in the West Bank of Palestine. Participants were allocated to CA-CBT (n = 15) or waitlist control (n = 15). The intervention comprised structured weekly brief psychotherapy sessions up to 12 individual sessions, adapted to the Palestinian sociocultural context. Panic symptoms were assessed using the Clinical Indicators Scale for Diagnosing Panic Disorder (CISDPD) and the Hamilton Anxiety Rating Scale (HAM-A), while quality of life was assessed using the WHOQOL-BREF. Analyses were conducted among participants who completed post-intervention assessments; therefore, outcome estimates should be interpreted as complete-case pilot findings. Thirty participants were enrolled, and 22 completed post-intervention assessment (CA-CBT: 13/15, 86.7%; waitlist: 9/15, 60.0%). Mixed-model repeated-measures ANOVA showed significant group × time interactions for CISDPD (F(1, 20) = 112.81, *p* < 0.001, partial η^2^ = 0.85) and WHOQOL-BREF (F(1, 20) = 10.67, *p* = 0.004, partial η^2^ = 0.35). The HAM-A group × time interaction was borderline and should be interpreted cautiously (F(1, 20) = 3.48, *p* = 0.05, partial η^2^ = 0.15). The findings provide feasibility evidence and preliminary outcome estimates to inform a fully powered randomized controlled trial with prespecified feasibility criteria, intention-to-treat analysis, treatment-fidelity assessment, and longer-term follow-up. Ethics approval: The study was approved by the Research Ethics Secretariat of Universiti Kebangsaan Malaysia (Approval No. JEP-2024-551). All participants received information about the study and provided written informed consent before participation. The study was not prospectively registered as a clinical trial.

## 1. Introduction

Mental health and quality of life are closely interconnected, as psychological distress can impair well-being, daily functioning, and life satisfaction (Pop et al., 2022). Accordingly, contemporary mental health care increasingly emphasizes functional recovery and quality of life alongside symptom reduction (Connell et al., 2014), particularly because mental health conditions may reduce autonomy, self-esteem, social participation, and daily functioning (Connell et al., 2012). Panic disorder is a disabling anxiety disorder associated with substantial psychological, social, and functional burden and poorer quality of life (Freidl et al., 2022; Wesner et al., 2019). Recurrent panic symptoms and avoidance can disrupt occupational, social, and family functioning and increase healthcare utilization, while comorbid psychiatric or medical conditions may further worsen health and treatment outcomes (Aquin et al., 2017; Manjunatha & Ram, 2022; Roca et al., 2023). When untreated, panic disorder may become persistent, contributing to long-term functional, educational, occupational, and financial difficulties (Manjunatha & Ram, 2022).

Cognitive behavioral therapy (CBT) is an established evidence-based intervention for anxiety disorders, demonstrating effectiveness in reducing panic symptoms and improving psychological functioning and quality of life (Wilmer et al., 2021; Pop et al., 2022). By reducing anxiety severity, agoraphobic avoidance, and somatic symptoms, CBT can contribute to broader improvements in quality of life and social, occupational, and emotional functioning (Pop et al., 2022; Ogawa et al., 2017). Importantly, improvements in quality of life may occur even without complete symptom remission (Rocha et al., 2020; Freidl et al., 2022). However, the effectiveness and acceptability of CBT may be influenced by cultural beliefs, values, social contexts, and treatment expectations, underscoring the need for culturally responsive adaptations across populations.

Despite strong evidence supporting CBT as a first-line treatment for panic disorder in Western contexts, its applicability and effectiveness in non-Western and conflict-affected settings remain insufficiently explored (Rathod et al., 2019). Cultural factors may influence how individuals understand, interpret, and communicate psychological and physical symptoms, including the meanings attributed to distress and the perceived causes of these experiences. Cultural, social, and family structures in Middle Eastern societies differ substantially from those commonly represented in Western psychological treatment models, with differences in traditions, social norms, community expectations, family dynamics, and beliefs about mental health potentially influencing help-seeking and engagement with psychotherapy (Boghosian, 2011; Hays & Abudabbeh, 2019). These considerations are particularly relevant in the Palestinian context, where psychological distress and mental health experiences occur within a complex sociocultural environment shaped not only by family and community structures but also by prolonged political conflict and related social, historical, and structural stressors (Jabr & Berger, 2023). Such contextual factors may influence how panic-related sensations are understood, expressed, and communicated, as well as how individuals perceive mental health treatment and engage with therapeutic interventions. However, evidence specifically examining the cultural presentation of panic disorder among Palestinians remains limited, and it is not yet clear whether standard panic-focused interventions fully address the cultural meanings, contextual stressors, communication patterns, and treatment needs relevant to this population (Akhtar et al., 2021). This represents an important gap because most psychotherapeutic models have been developed within Western contexts and may not fully correspond with the cultural values and contextual realities of Palestinian populations (Rathod et al., 2019). In Palestine, CBT and other psychological interventions remain limited and are often implemented without systematic coordination or full integration into mental health services, suggesting that simply transferring Western treatment models may not be sufficient without consideration of the local context (Jabr & Berger, 2023). Therefore, culturally adapting CBT requires attention to locally relevant symptom expression, illness beliefs, social and family norms, and factors that may influence patient engagement and treatment acceptability (Hinton & Patel, 2017; Algahtani et al., 2019). Accordingly, examining the potential clinical relevance of a culturally adapted CBT intervention for panic disorder among Palestinians represents an important step toward determining whether culturally and contextually informed modifications can enhance the applicability of the CA-CBT in this population.

Although CBT is effective for panic disorder, uncertainty remains regarding its delivery, cultural fit, and preliminary clinical effects in Palestinian settings. A pilot study is therefore warranted before a definitive efficacy trial. This study examined whether an individually delivered CA-CBT intervention was associated with preliminary changes in panic symptoms and quality of life, while also generating recruitment, retention, and attrition estimates to inform a future adequately powered randomized controlled trial.

## 2. Methodology

### 2.1. Study Design

This two-arm randomized pilot study compared an individually delivered culturally adapted cognitive behavioral therapy (CA-CBT) program with a waitlist control. The study was designed to estimate preliminary clinical effects and operational parameters rather than to provide definitive evidence of efficacy. Assessments were completed at baseline and immediately after the intervention or equivalent waiting period.

A waitlist control was selected to provide a contemporaneous comparison while ensuring that control participants could receive the intervention after the study period. Because waitlist controls may inflate apparent treatment effects relative to active controls, this design limitation was considered when interpreting the findings.

### 2.2. Participants and Setting

Eligible participants were adults aged 20–45 years with panic disorder diagnosed by a psychiatrist according to DSM-5 criteria. This age range was selected to capture the adult period in which panic disorder is commonly observed (Lijster et al., 2017; Olaya et al., 2018), while maintaining a relatively focused and homogeneous adult pilot sample. Participants were recruited from private psychiatric clinics in the Ramallah area of the West Bank, Palestine. Exclusion criteria included current psychosis, substance dependence, acute suicide risk requiring urgent clinical intervention, concurrent structured psychotherapy, and unstable medical illness that could interfere with safe participation or require immediate medical management, such as uncontrolled hypertension, unstable cardiac disease, or an acute neurological or medical condition.

Ramallah was selected because several psychiatric services were available and access was comparatively feasible during the study period. This pragmatic setting supported recruitment but limits generalizability to other Palestinian regions and to public-sector, rural, and conflict-intensified settings (Palestinian Central Bureau of Statistics, 2023).

### 2.3. Recruitment and Sampling Procedure

Participants were actively recruited from March through June 2025. Baseline assessments were completed in March 2025, following which the culturally adapted cognitive behavioral therapy (CA-CBT) intervention was delivered from March through June 2025. Post-intervention assessments were completed at the end of June 2025, marking the completion of the study.

Purposive recruitment was used to identify potentially eligible patients attending participating private psychiatric clinics. Recruitment notices invited interested individuals to contact the research team for screening. Eligible consenting participants were enrolled until the target sample of 30 was reached.

Following completion of the eligibility assessment and baseline measures, after enrollment, participants were assigned in a 1:1 ratio to CA-CBT or waitlist control using a lottery-based randomization procedure. Each participant had an equal probability of being assigned to either group. The lottery was conducted after baseline assessment to prevent knowledge of the allocation from influencing participant selection. The researcher responsible for implementing the intervention conducted the randomization, whereas outcome assessments were conducted separately. Because the study used a simple lottery procedure rather than a computerized randomization system, formal allocation concealment was limited. The extent to which outcome assessors were blinded to group allocation is reported explicitly as part of the study procedures.

### 2.4. Sample Size Rationale

The sample size was based on pilot-study feasibility rather than a formal power calculation (Teresi et al., 2022). A target of 30 participants was considered sufficient to estimate recruitment and retention and to obtain preliminary outcome estimates for planning a definitive trial (Whitehead et al., 2016). Accordingly, inferential results are exploratory, confidence intervals and effect-size precision are limited, and no definitive efficacy claim is warranted.

#### 2.4.1. Development and Cultural Adaptation of the Intervention

The cultural adaptation of cognitive behavioral therapy (CBT) for panic disorder in the West Bank of Palestine was conducted through a systematic, multi-stage process guided by the Southampton Adaptation Framework (SAF). First, a comprehensive literature review was undertaken to identify evidence-based CBT protocols for panic disorder and previous cultural adaptations of CBT within Arab and Muslim communities. Based on this review, an appropriate CBT intervention manual and adaptation framework were selected. The adaptation process then involved defining the target population, reviewing and selecting relevant intervention content, obtaining feedback from key stakeholders, incorporating proposed modifications, and subjecting the adapted program to professional and scientific review, including assessment of its content validity, before final refinement. The SAF guided the adaptation across three core domains: cultural awareness, assessment and engagement, and modification of therapeutic techniques. Stakeholder input was obtained through structured, recorded interviews using open-ended questions with 20 patients who had previously received CBT for panic disorder and 10 psychotherapists experienced in delivering CBT in Palestine. Patient interviews explored barriers to seeking treatment, experiences of CBT, clarity and cultural appropriateness of treatment content and terminology, challenges encountered during therapy, and the influence of therapist-related cultural, religious, and gender factors on treatment acceptability. Psychotherapist interviews examined the suitability of CBT techniques, culturally inappropriate components, implementation challenges, perceived benefits, patient beliefs and preferences, and recommendations for culturally responsive modifications. The qualitative data were analyzed thematically, and the findings were used to identify and refine culturally relevant modifications to the CBT intervention for Palestinian patients with panic disorder.

The CA-CBT protocol was developed from an established evidence-based CBT protocol for panic disorder that was developed by Barlow and colleagues for the treatment of panic disorder (Craske & Barlow, 2006). Core therapeutic components, including psychoeducation, cognitive restructuring, anxiety-management strategies, interoceptive and situational exposure, and relapse-prevention planning, were retained. The adaptations focused on language, examples, engagement strategies, explanatory models of panic symptoms, family and community context, stigma, accessibility, and chronic sociopolitical stressors relevant to Palestinian patients. These modifications were used to increase cultural relevance and acceptability while preserving the core mechanisms of CBT.

Accordingly, the intervention should be understood as culturally adapted CBT rather than a different therapeutic model. The adaptation was intended to improve fit, engagement, and applicability in the Palestinian clinical context, not to test whether cultural adaptation alone was responsible for clinical change (Table 1).

#### 2.4.2. Content Validation

Before feasibility testing and preliminary effectiveness evaluation, the adapted program underwent expert content validation. Six experts with experience in CBT for panic disorder were purposively selected; eligible experts held a master’s or doctoral degree in psychology or were psychiatrists with formal CBT training. Experts independently reviewed the program and completed a 25-item Content Validity Index (CVI) questionnaire covering definition, representation, relevance, and appropriateness of construction, using a four-point Likert scale from 1 (strongly disagree) to 4 (strongly agree). The results showed high content validity (CVI = 0.93).

### 2.5. Intervention Procedures

Participants received a structured, brief, individual psychotherapy program delivered once weekly for up to 12 sessions, with the intervention culturally adapted to the Palestinian sociocultural context, and each session lasting approximately 60 min. The intervention was delivered between 1 March and 20 June 2025 by the principal researcher. The 8-session core sequence covered psychoeducation, cognitive restructuring, anxiety-management strategies, interoceptive and situational exposure, and relapse-prevention planning. Additional sessions, up to a maximum of 12, were provided when participants required more time to complete exposure tasks, consolidate cognitive restructuring, or develop relapse-prevention strategies. Among participants who completed the CA-CBT intervention, attendance ranged from 8 to 10 sessions, with a mean of approximately 9.2 sessions. 

The principal researcher delivered the intervention program. Baseline and post-intervention outcome assessments were conducted in structured face-to-face interviews by an assistant psychologist who was not involved in delivering the intervention. Post-intervention assessments were intended to be blinded to group allocation, and participants were instructed not to disclose their allocation during assessment. Because the study was conducted in a routine clinical setting and blinding could not be independently verified, assessor blinding should be interpreted cautiously. All 30 participants completed baseline assessment, and 22 completed post-assessment (13 CA-CBT and 9 waitlist).

The intervention followed a structured therapeutic process consisting of:

1. Engagement and assessment: establishing the therapeutic relationship and identifying the participant’s main concerns.
2. Case formulation: identifying panic-related thoughts, emotions, behaviors, and contextual factors.
3. Goal setting: collaboratively establishing treatment goals.
4. Therapeutic intervention: delivering and practicing CBT techniques and coping strategies.
5. Evaluation and termination: reviewing treatment progress, consolidating skills, and developing relapse-prevention strategies.

The same adapted CBT protocol was used throughout the intervention period to promote consistency in treatment delivery.

### 2.6. Waitlist Control

Participants assigned to the waitlist control group completed the same baseline and post-intervention assessments but did not receive CA-CBT during the initial comparison period. To address ethical considerations, waitlist participants received monthly clinical evaluations during the waiting period. Where clinically indicated, waiting time could be adjusted to avoid unnecessarily prolonged withholding of treatment. After completion of the comparison period and post-test assessment, participants who remained eligible and expressed willingness to receive treatment were offered the CA-CBT program. Outcomes obtained after participants subsequently received CA-CBT were not included in the primary randomized comparison.

### 2.7. Concomitant Treatments

Participants were not permitted to receive concurrent structured psychotherapy for panic disorder from another provider during the study. The initiation of antidepressants, antipsychotics, anxiolytics, or other pharmacological treatment specifically intended to treat panic disorder symptoms during the intervention period was considered a protocol deviation. Changes in medication or other relevant treatments were documented.

### 2.8. Outcome Measures

Three outcome measures were used to assess panic disorder symptoms, anxiety severity, and quality of life. The study employed three established Arabic-language instruments with demonstrated psychometric support in Arabic-speaking populations: the Hamilton Anxiety Rating Scale (HAM-A), the Clinical Indications Scale for Diagnosing Panic Disorder (CISDPD), and the WHOQOL-BREF. The HAM-A is a clinician-administered 14-item scale assessing both psychological and somatic symptoms of anxiety and has been used to assess panic-related anxiety symptoms. The CISDPD, developed by Shoukair (2005) based on DSM-IV diagnostic criteria, consists of 30 Likert-scale items assessing panic disorder symptoms and has demonstrated satisfactory validity and reliability. The WHOQOL-BREF is a 26-item instrument assessing quality of life across physical health, psychological health, social relationships, and environmental domains, with strong psychometric properties and demonstrated validity in Arab populations (Dalky et al., 2016; Ohaeri & Awadalla, 2009; Rosén et al., 2020; WHO, 1997).

### 2.9. Blinding and Data Collection

Participants were assigned unique study identification codes to protect confidentiality and reduce the use of personally identifiable information during data handling. The CA-CBT intervention was delivered by the principal researcher, whereas baseline and post-intervention assessments were conducted by an assistant psychologist who was not involved in intervention delivery. The assessor was intended to remain unaware of participants’ group allocation, and participants were instructed not to disclose their allocation during assessment. Because the study was conducted in a routine clinical setting and assessor blinding could not be independently verified, the effectiveness of blinding was considered limited and is acknowledged as a methodological consideration.

### 2.10. Treatment Adherence and Retention

Attendance and treatment completion were monitored throughout the intervention. In the CA-CBT group, 13 of 15 participants (86.7%) completed the intervention, while two participants discontinued participation. Among completers, attendance ranged from 8 to 10 sessions, with a mean of approximately 9.2 sessions. In the waitlist group, 9 of 15 participants (60.0%) completed the comparison period, while six participants discontinued participation. Monthly clinical monitoring contacts were offered during the waiting period, but these contacts were not treated as psychotherapy sessions and were not included as intervention exposure.

### 2.11. Ethical Considerations

Participants in the waitlist group received monthly clinical monitoring during the waiting period, and earlier clinical intervention was permitted when clinically indicated. Treatment changes were recorded during the study; one participant in the CA-CBT group initiated antidepressant medication during the intervention phase and was excluded from the final complete-case analysis according to the study protocol.

After the comparison period and post-test assessment, all waitlist participants who remained eligible were offered the full CA-CBT intervention, ensuring equitable access to treatment.

Outcomes collected after subsequent waitlist treatment were not included in the present randomized comparison.

### 2.12. Statistical Analysis

Data were analyzed using SPSS version 23. Participant characteristics and retention were summarized using frequencies, percentages, means, and standard deviations. Preliminary clinical outcomes were examined using paired comparisons within groups and mixed-model repeated-measures analysis of variance for group, time, and group-by-time effects. Because analyses included only participants with post-intervention data, they represent complete-case analyses and may be vulnerable to attrition bias. Exact *p* values are reported where available; values produced as 0.000 by SPSS are reported as *p* < 0.001. Effect sizes are presented as partial eta squared (partial η^2^).

## 3. Results

### 3.1. Participant Characteristics

There were no statistically significant differences between the treatment and waitlist groups in terms of age, gender, or educational level at baseline (all *p* > 0.05), indicating that the groups were generally comparable with respect to the measured demographic characteristics (Table 2).

### 3.2. Recruitment, Retention, and Participant Flow

**Figure 1:**
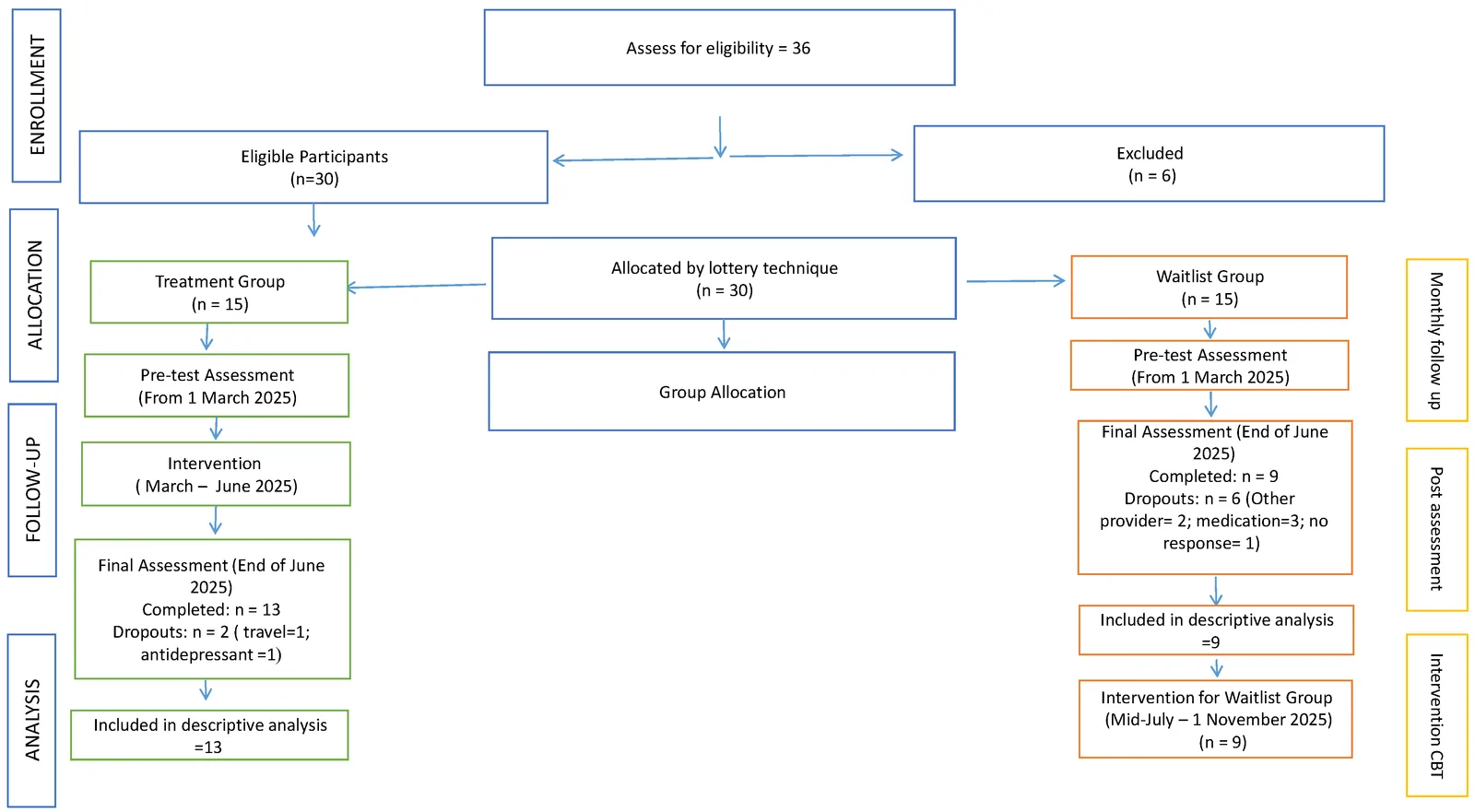
CONSORT flow diagram of participant recruitment, allocation, follow-up, and analysis.

Participants were recruited and screened between March and June 2025. Thirty eligible participants were randomized: 15 to CA-CBT and 15 to waitlist control. Recruitment, allocation, follow-up, and analysis are summarized in a CONSORT-style flow diagram (see Figure 1).

### 3.3. Preliminary Clinical Outcomes

Preliminary outcomes were panic symptom severity, measured using the CISDPD and HAM-A, and quality of life, measured using the WHOQOL-BREF. Outcomes were assessed at baseline and post-intervention or post-waiting period.

Shapiro–Wilk tests did not provide evidence of substantial departures from normality (W = 0.886–0.976, *p* = 0.051–0.939), and Levene’s tests did not indicate unequal variances (F = 0.02–3.57, all *p* > 0.05). These tests have limited power in small samples; therefore, graphical inspection and robust or nonparametric sensitivity analyses would strengthen a future report.

Mixed-model repeated-measures ANOVA was used to examine time, group, and group-by-time effects for CISDPD, HAM-A, and WHOQOL-BREF scores (Table 3).

Within the CA-CBT group, CISDPD scores decreased from M = 2.24 (SD = 0.41) at baseline to M = 0.38 (SD = 0.27) post-intervention (*p* < 0.001). The waitlist group showed little change, from M = 1.43 (SD = 0.68) to M = 1.33 (SD = 0.56; *p* = 0.32).

HAM-A scores in the CA-CBT group decreased from M = 2.58 (SD = 0.72) to M = 0.64 (SD = 0.50; *p* < 0.001). The waitlist group showed no evidence of improvement, changing from M = 2.02 (SD = 0.74) to M = 2.11 (SD = 0.60; *p* = 0.34).

WHOQOL-BREF scores in the CA-CBT group increased from M = 2.82 (SD = 0.65) to M = 3.79 (SD = 0.56; *p* < 0.001). The waitlist group changed from M = 2.97 (SD = 0.41) to M = 2.88 (SD = 0.28; *p* = 0.22) (Table 4).

For CISDPD, the group-by-time interaction was statistically significant, F(1, 20) = 112.81, *p* < 0.001, partial η^2^ = 0.85, indicating a substantially larger reduction in the CA-CBT group than in the waitlist group. The main effects of time and group were also significant; however, in a randomized pre–post study, the interaction is the most relevant test of differential change.

For HAM-A, the main effect of time was significant, F(1, 20) = 228.50, *p* < 0.001, partial η^2^ = 0.92, indicating an overall reduction in anxiety symptoms from pre- to post-test. The group-by-time interaction was borderline, F(1, 20) = 3.48, *p* = 0.05, partial η^2^ = 0.15, and should therefore be interpreted cautiously rather than as definitive evidence of differential treatment effect.

For WHOQOL-BREF, the group-by-time interaction was significant, F(1, 20) = 10.67, *p* = 0.004, partial η^2^ = 0.35, indicating greater improvement in the CA-CBT group. The large effect-size estimates across outcomes may be unstable because of the small complete-case sample and should be accompanied by confidence intervals in future analyses.

## 4. Discussion

This randomized pilot study found promising reductions in panic symptoms and improvement in quality of life among participants who completed CA-CBT compared with those who completed the waitlist period. The clearest evidence of differential change was observed for CISDPD and WHOQOL-BREF. The HAM-A group-by-time effect was at the conventional significance threshold and therefore warrants more cautious interpretation.

The direction of the findings is consistent with evidence supporting CBT for panic disorder across face-to-face and remote formats (Efron & Wootton, 2021) and with previous evidence suggesting that culturally adapted psychological interventions may improve outcomes across diverse cultural groups (Hall et al., 2016; Soto et al., 2018; Arundell et al., 2021). The present study extends this literature by testing an intervention adapted to Palestinian language, explanatory models, family and community influences, stigma, and chronic sociopolitical stressors. However, the waitlist comparison does not adequately control for therapist attention, treatment expectancy, therapeutic contact, or other nonspecific treatment effects, and evidence suggests that psychotherapy effects may be larger when compared with waitlist controls than with active controls (Furukawa et al., 2014).

Although the core therapeutic mechanisms of CBT for panic disorder, including psychoeducation, cognitive restructuring, and exposure-based procedures, were retained, their content and delivery were adapted to the sociocultural and contextual experiences of Palestinian participants. This included the use of locally understandable Arabic terminology and examples, exploration of culturally shaped illness beliefs and stigma, consideration of family and community contexts, and integration of chronic conflict-related stressors, movement restrictions, and culturally meaningful coping resources into the therapeutic process. Such adaptations are consistent with established frameworks of culturally adapted CBT, which emphasize that adaptation may involve changes in cultural understanding, assessment and engagement, practical delivery, therapeutic techniques, and the contextual framing of psychological difficulties (Rathod et al., 2019; Naeem et al., 2019). Importantly, the present findings do not establish that these adaptations themselves caused the observed clinical improvements, because the study did not directly compare culturally adapted CBT with an otherwise equivalent non-adapted CBT intervention. Rather, the findings provide preliminary evidence that an adapted CBT/PCT approach can be feasibly delivered and may be clinically relevant for Palestinian adults experiencing panic symptoms. The adaptation may be particularly relevant in settings where psychological symptoms occur alongside persistent environmental stressors and where treatment engagement can be influenced by cultural beliefs, stigma, family relationships, and practical barriers to accessing care. Future trials should therefore directly compare culturally adapted and standard CBT, while examining whether specific adaptations improve treatment acceptability, engagement, retention, and clinical outcomes. Such research would help determine whether cultural adaptation provides benefits beyond those achieved by standard evidence-based CBT and identify which components are most important for Palestinian populations.

Previous research supports the effectiveness of brief CBT for panic disorder (Samantaray et al., 2022). However, the present pilot study should not be interpreted as equivalent to larger efficacy trials. Its contribution is preliminary: it demonstrates that CA-CBT could be delivered in a routine Palestinian clinical setting and provides outcome and attrition estimates for a future trial.

Quality of life improved in the CA-CBT completer group, consistent with research showing that reductions in panic, agoraphobic avoidance, and anxiety-related somatic symptoms may translate into better functioning and well-being. Because the WHOQOL-BREF is broad, future studies should examine which domains improve most and whether gains are maintained beyond immediate post-treatment. These findings are consistent with Bilet et al. (2020), who reported that CBT improved health-related and overall quality of life among individuals with panic disorder.

The findings should also be interpreted in light of differential attrition. Retention was higher in the CA-CBT group than in the waitlist group, and several waitlist participants sought alternative treatment or medication. This differential attrition may have introduced selection bias and may have resulted in a more stable subgroup being available for follow-up. Missing outcome data can compromise the validity of treatment-effect estimates, particularly when dropout differs between randomized groups (Little et al., 2012; White et al., 2011). In addition, the use of a waitlist condition may contribute to differential dropout because participants receiving no immediate treatment may seek alternative care (Furukawa et al., 2014). Although mixed-effects models and multiple imputation can provide more appropriate approaches to missing data under plausible assumptions, their use should be prespecified and accompanied by sensitivity analyses (Little et al., 2012; White et al., 2011). Future definitive trials should therefore adopt an intention-to-treat framework and appropriate methods for handling missing data.

Overall, CA-CBT showed preliminary improvements on panic symptoms and quality of life in this setting. However, given the pilot design, small sample size, differential attrition, and use of a waitlist control, these findings should be considered preliminary and do not establish treatment effectiveness. Further evaluation in a larger, adequately powered randomized controlled trial is warranted, incorporating concealed allocation, blinded outcome assessment where feasible, an active comparison condition, prespecified primary and feasibility outcomes, intervention-fidelity monitoring, systematic adverse-event reporting, and longer-term follow-up (Schulz et al., 2010; Eldridge et al., 2016; Bellg et al., 2004).

## 5. Strengths and Limitations

This study addresses an under-researched clinical problem by evaluating a culturally adapted psychological intervention for panic disorder within a routine Palestinian clinical setting. Key strengths include randomized allocation; independent outcome assessment; the use of validated measures of panic symptoms, anxiety, and quality of life; and transparent reporting of attrition and reasons for loss to follow-up. The study also provides preliminary evidence on the feasibility and clinical relevance of delivering a culturally adapted intervention in a setting that is underrepresented in the psychological intervention literature.

Important limitations include the small sample, single geographic setting, private-clinic recruitment, short follow-up, therapist delivery by the principal researcher, uncertain assessor blinding, variable session dose, absence of an active control, and complete-case analysis. Differential attrition was substantial, particularly in the waitlist group, and one post-randomization exclusion occurred after medication initiation. These factors limit causal inference, precision, and generalizability.

Future studies should prospectively register the protocol, define feasibility progression criteria, report intervention fidelity and adherence, use intention-to-treat analysis, provide confidence intervals for effect estimates, assess clinical significance in addition to statistical significance, and evaluate maintenance of gains over longer follow-up.

## 6. Conclusions

This pilot study demonstrated the feasibility of recruiting and delivering a culturally adapted CBT intervention for panic disorder within Palestinian clinical settings. Recruitment targets were met, with 30 participants enrolled within the planned recruitment period, and overall post-assessment retention reached 73.3%, providing useful feasibility information for planning a definitive trial. Retention in the intervention group was particularly favorable (86.7%), supporting the feasibility and acceptability of treatment delivery. However, the lower retention in the waitlist group and the reported use of alternative treatment highlight the need for strengthened retention procedures in future trials.

Preliminary outcome findings suggest improvements in panic symptoms and quality of life following CA-CBT; however, these findings should be interpreted cautiously given the small sample, waitlist comparison, differential attrition, short follow-up, and complete-case analysis. The study therefore provides feasibility evidence and preliminary estimates rather than definitive evidence of efficacy. A fully powered, prospectively registered randomized controlled trial is warranted to evaluate efficacy and longer-term durability, while incorporating multiple therapists and service settings, appropriate missing-data methods, intervention-fidelity monitoring, and an active comparison condition to better distinguish the specific effects of cultural adaptation from nonspecific treatment effects.

## Figures and Tables

**Table 1:** Cultural adaptation matrix of the modified CBT components based on the cultural adaptation phase.

Domain/CBT Component	Culturally Adapted Intervention for Palestinian Participants	Rationale/Intended Contribution
Language and communication	Use simple, locally understood Arabic expressions and culturally appropriate terminology; replace technical psychological terms with everyday language.	Improves comprehension, therapeutic communication, and participant engagement.
Manual, worksheets, and therapist scripts	Adapted the language, examples, scenarios, and explanatory materials to Palestinian cultural and everyday contexts rather than directly translating standard CBT materials.	Improves cultural relevance, comprehension, and engagement with homework and therapeutic exercises.
Political conflict, chronic stress, and daily adversity	Discussion of chronic stress related to occupation, insecurity, movement restrictions, family responsibilities, and daily adversity was incorporated when relevant to the participant’s presentation.	Allows panic symptoms to be understood within the participant’s actual environment rather than assuming that all anxiety-related concerns reflect individual cognitive distortions.
Case formulation and cognitive restructuring	Cognitive formulation incorporated participants’ lived experiences, family context, social circumstances, and realistic environmental concerns. Therapists distinguished realistic concerns about external threats from catastrophic misinterpretations of internal panic sensations.	Prevents culturally and contextually appropriate concerns from being incorrectly conceptualized as maladaptive cognitions and enhances treatment relevance.
Cultural concepts and beliefs about mental disorder	Assessment and therapy explored culturally shaped beliefs about anxiety, panic, bodily symptoms, mental illness, and help-seeking. Locally meaningful expressions such as “tightness in the chest” and “fear of dying” were incorporated when describing panic experiences.	Improves communication, facilitates accurate understanding of symptom meaning, and increases cultural acceptability.
Assessment and psychoeducation	Psychoeducation about panic and the fight-or-flight response was delivered using culturally familiar examples and Arabic terminology and was linked to participants’ everyday experiences.	Enhances understanding of panic symptoms and reduces misunderstanding of culturally expressed psychological distress.
Mental health stigma	Non-stigmatizing language such as “stress,” “anxiety,” and “well-being” was used where appropriate, and concerns regarding stigma and help-seeking were openly discussed.	Reduces barriers to psychological treatment and supports engagement and acceptance.
Psychoeducation, communication, and cognitive restructuring	Standard CBT principles were retained, but explanations and cognitive restructuring exercises were adapted to participants’ cultural beliefs, family context, and experiences of chronic stress.	Preserves the core therapeutic mechanism while increasing cultural relevance and acceptability.
Interoceptive exposure	Interoceptive exposure was retained as a core PCT component but was introduced using culturally understandable explanations of feared bodily sensations. Participants’ beliefs about symptoms such as palpitations, dizziness, shortness of breath, and loss of control were explored before exposure.	Maintains the evidence-based mechanism of exposure while addressing culturally shaped interpretations and fears of bodily sensations.
Situational exposure	Exposure tasks were collaboratively selected according to participants’ actual avoidance patterns and environmental circumstances. Panic-related avoidance was distinguished from avoidance of objectively unsafe situations.	Maintains the therapeutic function of exposure while ensuring that treatment does not disregard genuine safety concerns.
Accessibility and service delivery	Flexible scheduling and, when necessary and feasible, online sessions were offered when travel or movement restrictions created barriers to attendance.	Improves accessibility, continuity, and treatment attendance.
Therapist cultural competence	Therapists considered local cultural norms, family relationships, social expectations, religious considerations, stigma, and the sociopolitical context when delivering the intervention.	Supports culturally responsive, respectful, and contextually appropriate treatment delivery.
Problem solving and stress management	Problem-solving and stress-management strategies were incorporated when ongoing contextual stressors were contributing to or exacerbating panic symptoms, while maintaining the primary therapeutic focus on panic disorder.	Helps participants manage modifiable contextual stressors without replacing the core panic-focused CBT intervention.
Interpersonal and family context	Where relevant, family and interpersonal relationships were incorporated into formulation and problem solving, recognizing the importance of family and community support in participants’ daily lives.	Enhances contextual relevance and identifies available sources of social support.
Culturally meaningful coping and resilience	Culturally meaningful coping resources, including the concept of *Sumud* (steadfastness/resilience), were explored when relevant to participants’ coping experiences.	Builds on culturally meaningful sources of resilience and strengthens the relevance of coping strategies.
Peer support	A peer-support component was incorporated to provide an opportunity for participants to share experiences, normalize panic-related difficulties, and discuss coping strategies in a supportive environment.	Reduces isolation, normalizes experiences, provides social support, and may enhance self-efficacy and treatment engagement.

**Table 2:** Baseline demographic characteristics by randomized group.

Variable	Levels	Total (N = 30)	Waitlist (N = 15)	Treatment (N = 15)	*p*-Value
Age (years)	20–25	6 (20.0)	2 (13.3)	4 (26.7)	**0** **.172**
	>25–30	12 (40.0)	9 (60.0)	3 (20.0)	
	>30–35	6 (20.0)	2 (13.3)	4 (26.7)	
	>35	6 (20.0)	2 (13.3)	4 (26.7)	
Gender	Male	19 (63.3)	9 (60.0)	10 (66.7)	**1.000**
	Female	11 (36.7)	6 (40.0)	5 (33.3)	
Education	Master’s	2 (6.7)	2 (13.3)	0 (0.0)	**0** **.289**
	Bachelor’s	20 (66.7)	9 (60.0)	11 (73.3)	
	Less than Bachelor’s	8 (26.7)	6 (40.0)	4 (26.7)	

**Table 3:** Descriptive statistics and paired pre–post comparisons for CISDPD, HAM-A, and WHOQOL-BREF.

Measure	Group	N	Pre-Mean ± SD	Post-Mean ± SD	*t*-Test	*p* Value
CISDPD	Treatment	13	2.24 ± 0.41	0.38 ± 0.27	15.19	<0.001
	Waitlist	9	1.43 ± 0.68	1.33 ± 0.56	1.06	0.32
	Total	22	1.91 ± 0.66	0.77 ± 0.62	—	—
HAM-A	Treatment	13	2.58 ± 0.72	0.64 ± 0.50	8.98	<0.001
	Waitlist	9	2.02 ± 0.74	2.11 ± 0.60	−1.01	0.34
	Total	22	2.35 ± 0.76	1.24 ± 0.91	—	—
WHOQOL-BREF	Treatment	13	2.82 ± 0.65	3.79 ± 0.56	−3.70	<0.001
	Waitlist	9	2.97 ± 0.41	2.88 ± 0.28	1.34	0.22
	Total	22	2.88 ± 0.56	3.42 ± 0.65	—	—

**Table 4:** Mixed-model repeated-measures ANOVA for CISDPD, HAM-A, and WHOQOL-BREF.

Measure	Source of Variation	Type III SS	df	Mean Square	F	*p* Value	Partial Eta^2^
Panic (CISDPD)	Between-Subjects						
	Intercept	0.05	1	0.05	0.13	0.72	0.01
	Group	76.78	1	76.78	204.15	<0.001	0.91
	Error	7.52	20	0.38	—	—	—
	Within-Subjects						
	Panic (Pre–Post)	10.22	1	10.22	138.73	<0.001	0.87
	Panic × Group	8.31	1	8.31	112.81	<0.001	0.85
	Error	1.47	20	0.07	—	—	—
HAM-A	Between-Subjects						
	Intercept	8.99	1	8.99	45.63	<0.001	0.70
	Group	10.95	1	10.95	55.57	<0.001	0.74
	Error	3.94	20	0.20	—	—	—
	Within-Subjects						
	Hamilton (Pre–Post)	143.52	1	143.52	228.50	<0.001	0.92
	Hamilton × Group	2.19	1	2.19	3.48	0.05	0.15
	Error	12.56	20	0.63	—	—	—
WHOQOL-BREF	Between-Subjects						
	Intercept	413.21	1	413.21	1529.56	<0.001	0.99
	Group	1.56	1	1.56	5.76	0.03	0.22
	Error	5.40	20	0.27	—	—	—
	Within-Subjects						
	Quality of Life (Pre–Post)	2.10	1	2.10	7.63	0.01	0.28
	Quality of Life × Group	2.94	1	2.94	10.67	<0.001	0.35
	Error	5.51	20	0.28	—	—	—

## Data Availability

The raw data supporting the conclusions of this article will be made available by the authors on request.
